# Supplementary material for: Multi‐dataset identification of innovative feature genes and molecular mechanisms in keratoconus
Source: J Cell Mol Med. 2024 Sep 19;28(18):e70079. doi: 10.1111/jcmm.70079 (PMC11412914; doi:10.1111/jcmm.70079)
Supplement: Supplementary file 2 — Material S2. [file JCMM-28-e70079-s002.zip › 7.GSEA analysis/4.MYRF/gsea_report_for_h_1678852873579.html]

Report for h 1678852873579 [GSEA]

| GS  follow link to MSigDB | GS DETAILS | SIZE | ES | NES | NOM p-val | FDR q-val | FWER p-val | RANK AT MAX | LEADING EDGE || 1 | KEGG\_PORPHYRIN\_AND\_CHLOROPHYLL\_METABOLISM | Details ... | 32 | 0.44 | 1.40 | 0.083 | 1.000 | 0.884 | 7657 | tags=25%, list=22%, signal=32% |
| 2 | KEGG\_TERPENOID\_BACKBONE\_BIOSYNTHESIS | Details ... | 15 | 0.44 | 1.20 | 0.277 | 1.000 | 0.987 | 12132 | tags=60%, list=35%, signal=92% |
| 3 | KEGG\_TASTE\_TRANSDUCTION | Details ... | 52 | 0.39 | 1.12 | 0.285 | 1.000 | 0.997 | 3193 | tags=25%, list=9%, signal=27% |
| 4 | KEGG\_CYSTEINE\_AND\_METHIONINE\_METABOLISM | Details ... | 34 | 0.25 | 1.10 | 0.322 | 1.000 | 0.998 | 7233 | tags=21%, list=21%, signal=26% |
| 5 | KEGG\_HOMOLOGOUS\_RECOMBINATION | Details ... | 26 | 0.32 | 1.06 | 0.416 | 1.000 | 0.999 | 1592 | tags=8%, list=5%, signal=8% |
| 6 | KEGG\_GLYCOSAMINOGLYCAN\_BIOSYNTHESIS\_HEPARAN\_SULFATE | Details ... | 26 | 0.30 | 1.02 | 0.404 | 1.000 | 1.000 | 7349 | tags=27%, list=21%, signal=34% |
| 7 | KEGG\_RNA\_DEGRADATION | Details ... | 51 | 0.19 | 1.01 | 0.497 | 1.000 | 1.000 | 27979 | tags=100%, list=81%, signal=514% |
| 8 | KEGG\_GLYCOSAMINOGLYCAN\_DEGRADATION | Details ... | 21 | 0.30 | 0.95 | 0.493 | 1.000 | 1.000 | 3619 | tags=10%, list=10%, signal=11% |
| 9 | KEGG\_GLYCOSPHINGOLIPID\_BIOSYNTHESIS\_GANGLIO\_SERIES | Details ... | 15 | 0.33 | 0.94 | 0.542 | 1.000 | 1.000 | 13266 | tags=60%, list=38%, signal=97% |
| 10 | KEGG\_OOCYTE\_MEIOSIS | Details ... | 111 | 0.18 | 0.92 | 0.573 | 1.000 | 1.000 | 8743 | tags=20%, list=25%, signal=26% |
| 11 | KEGG\_CITRATE\_CYCLE\_TCA\_CYCLE | Details ... | 30 | 0.22 | 0.90 | 0.551 | 1.000 | 1.000 | 2914 | tags=7%, list=8%, signal=7% |
| 12 | KEGG\_STEROID\_BIOSYNTHESIS | Details ... | 17 | 0.28 | 0.89 | 0.579 | 1.000 | 1.000 | 12032 | tags=59%, list=35%, signal=90% |
| 13 | KEGG\_PROTEIN\_EXPORT | Details ... | 22 | 0.27 | 0.87 | 0.699 | 1.000 | 1.000 | 25245 | tags=100%, list=73%, signal=366% |
| 14 | KEGG\_MISMATCH\_REPAIR | Details ... | 23 | 0.24 | 0.84 | 0.744 | 1.000 | 1.000 | 616 | tags=4%, list=2%, signal=4% |
| 15 | KEGG\_ASCORBATE\_AND\_ALDARATE\_METABOLISM | Details ... | 17 | 0.30 | 0.83 | 0.738 | 1.000 | 1.000 | 7657 | tags=29%, list=22%, signal=38% |
| 16 | KEGG\_GLYCEROLIPID\_METABOLISM | Details ... | 43 | 0.23 | 0.82 | 0.790 | 1.000 | 1.000 | 9065 | tags=35%, list=26%, signal=47% |
| 17 | KEGG\_PENTOSE\_AND\_GLUCURONATE\_INTERCONVERSIONS | Details ... | 20 | 0.30 | 0.81 | 0.749 | 1.000 | 1.000 | 7657 | tags=25%, list=22%, signal=32% |
| 18 | KEGG\_AMINOACYL\_TRNA\_BIOSYNTHESIS | Details ... | 22 | 0.26 | 0.79 | 0.709 | 1.000 | 1.000 | 25608 | tags=100%, list=74%, signal=381% |
| 19 | KEGG\_OLFACTORY\_TRANSDUCTION | Details ... | 372 | 0.23 | 0.77 | 0.996 | 1.000 | 1.000 | 7634 | tags=32%, list=22%, signal=41% |
| 20 | KEGG\_ALANINE\_ASPARTATE\_AND\_GLUTAMATE\_METABOLISM | Details ... | 30 | 0.23 | 0.76 | 0.815 | 1.000 | 1.000 | 5643 | tags=17%, list=16%, signal=20% |
| 21 | KEGG\_STARCH\_AND\_SUCROSE\_METABOLISM | Details ... | 39 | 0.24 | 0.73 | 0.810 | 1.000 | 1.000 | 6521 | tags=21%, list=19%, signal=25% |
| 22 | KEGG\_PEROXISOME | Details ... | 78 | 0.18 | 0.70 | 0.852 | 1.000 | 1.000 | 10709 | tags=29%, list=31%, signal=43% |
| 23 | KEGG\_FATTY\_ACID\_METABOLISM | Details ... | 42 | 0.20 | 0.66 | 0.889 | 1.000 | 1.000 | 5377 | tags=14%, list=15%, signal=17% |
| 24 | KEGG\_PRIMARY\_IMMUNODEFICIENCY | Details ... | 35 | 0.24 | 0.66 | 0.936 | 1.000 | 1.000 | 5912 | tags=14%, list=17%, signal=17% |
| 25 | KEGG\_MATURITY\_ONSET\_DIABETES\_OF\_THE\_YOUNG | Details ... | 25 | 0.23 | 0.62 | 0.990 | 1.000 | 1.000 | 8376 | tags=32%, list=24%, signal=42% |
| 26 | KEGG\_GLYCOSYLPHOSPHATIDYLINOSITOL\_GPI\_ANCHOR\_BIOSYNTHESIS | Details ... | 25 | 0.17 | 0.52 | 0.956 | 1.000 | 1.000 | 28916 | tags=100%, list=83%, signal=597% |
| 27 | KEGG\_PENTOSE\_PHOSPHATE\_PATHWAY | Details ... | 27 | 0.13 | 0.50 | 1.000 | 0.990 | 1.000 | 11079 | tags=30%, list=32%, signal=43% |
Table: Gene sets enriched in phenotype **h (8 samples) **[plain text format]****
